# Virtual (Computed) Fractional Flow Reserve: Future Role in Acute Coronary Syndromes

**DOI:** 10.3389/fcvm.2021.735008

**Published:** 2021-10-22

**Authors:** Hazel Arfah Haley, Mina Ghobrial, Paul D. Morris, Rebecca Gosling, Gareth Williams, Mark T. Mills, Tom Newman, Vignesh Rammohan, Giulia Pederzani, Patricia V. Lawford, Rodney Hose, Julian P. Gunn

**Affiliations:** ^1^Department of Infection Immunity and Cardiovascular Disease, University of Sheffield, Sheffield, United Kingdom; ^2^Insigneo Institute for in silico Medicine, Sheffield, United Kingdom; ^3^Sheffield Teaching Hospitals National Health Service Foundation Trust, Sheffield, United Kingdom

**Keywords:** computed blood flow, ACS - ACS/NSTEMI, FFR, vFFR, virtual FFR, angiogram based FFR, coronary artery modelling

## Abstract

The current management of acute coronary syndromes (ACS) is with an invasive strategy to guide treatment. However, identifying the lesions which are physiologically significant can be challenging. Non-invasive imaging is generally not appropriate or timely in the acute setting, so the decision is generally based upon visual assessment of the angiogram, supplemented in a small minority by invasive pressure wire studies using fractional flow reserve (FFR) or related indices. Whilst pressure wire usage is slowly increasing, it is not feasible in many vessels, patients and situations. Limited evidence for the use of FFR in non-ST elevation (NSTE) ACS suggests a 25% change in management, compared with traditional assessment, with a shift from more to less extensive revascularisation. Virtual (computed) FFR (vFFR), which uses a 3D model of the coronary arteries constructed from the invasive angiogram, and application of the physical laws of fluid flow, has the potential to be used more widely in this situation. It is less invasive, fast and can be integrated into catheter laboratory software. For severe lesions, or mild disease, it is probably not required, but it could improve the management of moderate disease in 'real time' for patients with non-ST elevation acute coronary syndromes (NSTE-ACS), and in bystander disease in ST elevation myocardial infarction. Its practicability and impact in the acute setting need to be tested, but the underpinning science and potential benefits for rapid and streamlined decision-making are enticing.

## What Are the Issues With Coronary Angiography in ACS?

The fundamental limitation of CAG is that it is an anatomical, not a physiological, test which reveals luminal stenoses, with a poor relationship to blood flow and the identification of “significant” coronary artery disease (1). The severity of an angiographic lesion is typically over-estimated by eye, and the length under-estimated (2). There is also considerable inter-observer variability in lesion assessment (3). These weaknesses are only partly addressed by using quantitative coronary angiography, which has its own limitations (4). In addition, assessment is critically dependent upon the quality of the angiographic images; inadequate contrast, insufficient projections, overlapping vessels, excess movement, and lesions located at ostia, branch points and in series pose particular challenges. In addition, it cannot reveal the vulnerability or instability of lesions without the assistance of intravascular imaging, although this is also a limitation of physiological assessment (5).

## What Is Physiological Guidance?

**Figure 1:**
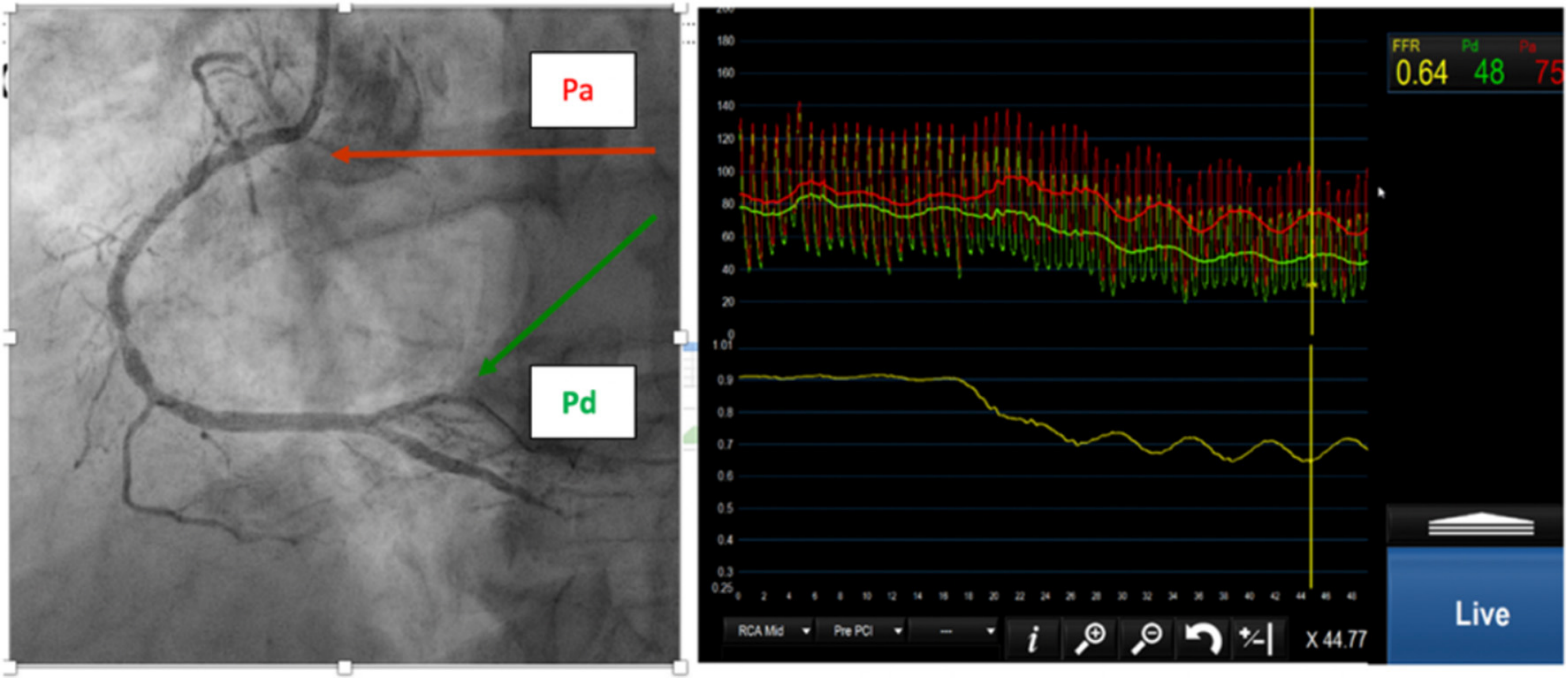
**(Left)** Right coronary angiogram with moderate stenoses. **(Right)** Proximal (red) and distal (green) pressure. The FFR is 0.64.

Fractional flow reserve (FFR) is the ratio of the maximum achievable blood flow in the stenotic coronary artery to the theoretical maximum flow in an equivalent normal coronary artery:


$$\begin{array}{l} FFR\;=\;\frac{Q\;with\;stenosis}{Q\;normal\;}\;=\;\frac{\left(\left(P_{d}-P_{v}\right)/R\right)}{\left(\left(P_{a}-P_{v}\right)/R\right)}\;\approx \;\frac{P_{d}}{P_{a}} \end{array}$$

where Pa is mean aortic (proximal) pressure, Pd is pressure distal to the stenosis, Pv is the central venous pressure and R is resistance to flow. FFR approximates to the ratio of the distal to proximal pressure, so it can be measured with a pressure-sensitive angioplasty guidewire. FFR is best calculated during maximum hyperaemia, during which microvascular resistance (MVR) is assumed minimal or constant which can be achieved by an infusion of adenosine (see Figure 1).

FFR, or related 'resting' indices such as resting full cycle ratio (RFR) or instantaneous wave-free ratio (iFR), is recommended in arteries with narrowing estimated visually between 50 and 90%, when non-invasive testing is unavailable or inconclusive (6). An FFR < 0.80 is the accepted threshold for ischaemia and justifies intervention. Physiological guidance, compared with angiography alone, reduces symptom burden, repeat revascularisation and health expenditure at the time of percutaneous coronary intervention (PCI) (7–9). A hidden benefit of physiological guidance is that, perhaps unfortunately, angiographic precision is not essential.

## What Is the Evidence for FFR in NSTE-ACS?

Whilst the majority of the evidence for FFR is in chronic coronary syndromes (CCS), there were large NSTE-ACS subsets in some of the seminal studies. In the FAME study, 30% of patients had NSTE-ACS. The 2-year rate of major adverse cardiovascular events (MACE) was significantly reduced in the FFR- vs. the CAG-guided groups, with no difference between the CCS and NSTE-ACS cohorts (10). Importantly, the study also showed that no myocardial infarctions (MI)s occurred in the FFR-guided deferred lesions in the NSTE-ACS cohort at 2 year follow up (11). A health economic analysis from the study also revealed that FFR-guided PCI improved outcomes and costs at 1 year when compared with the CAG-guided approach (12). In a “real-world” observational study of 3,000 patients with ACS, a lower in-hospital mortality was observed for FFR guidance than for a CAG-based approach (1.1 vs. 3.1%, *p* < 0.01), and reductions in hospital stay, acute kidney injury (AKI) and bleeding (13). In a study of 350 ACS patients randomised to FFR- vs. CAG-guidance, disclosure of the FFR resulted in changed management in 21.6% of cases, reducing the number of unnecessary procedures and downstream unplanned revascularizations (14). A cost-effectiveness assessment disclosed that increased up-front costs (pressure wire use and laboratory time) were more than compensated by later savings in subsequent hospital stay, events and procedures; and there was also a small benefit in quality-adjusted life year (QALYs) (15). In another study of 107 patients with multi-vessel disease and moderate non-culprit lesions, FFR resulted in 76% of patients not being revascularised; and importantly there was no MACE in this group (16). A meta-analysis of the three major RCTs also concluded that FFR guidance in patients with NSTE-ACS led to a reduction in the rate of MI without any difference in death or all-cause mortality and target vessel revascularisation compared with CAG guided approach (17). In a study of 1,983 patients with ACS (*n* = 533) and CCS (*n* = 1,450), FFR led to a similarly high percentage of reclassification of treatment in both groups (ACS = 38% vs. CCS = 39%). In the ACS patients, FFR guidance led to a change from revascularization in 70% and medical therapy in 30% to revascularisation in 38% and medical therapy in 62%. There was no significant difference in MACE (8.0 vs. 11.6%; *p* = 0.20) or symptoms (92.3 vs. 94.8% angina free; *p* = 0.25) between the reclassified (FFR discordant with CAG) vs. the non-reclassified patients (FFR concordant with CAG) groups. FFR-guided deferral to medical therapy in the ACS group was as safe as in the CCS group (MACE 8 vs. 8.5%; revascularization 3.8 vs. 5.9%; and freedom from angina 93.6 vs. 90.2%). Worse outcomes were observed in the six percent of patients in whom FFR was disregarded (18). In a study of 1,596 patients of which 301 had ACS (*n* = 449 lesions), deferral of the non-culprit lesion based upon FFR resulted in a MACE 3.8% (ACS) vs. 1.6% (CCS), mainly driven by ischaemia-driven revascularisation (2.8 vs. 1.1%) (19). Two systematic reviews comparing available data on FFR guidance confirmed this difference, with no significant difference in mortality (20, 21). ESC guidelines propose that FFR can be used in ACS (class IIb) to assist decision-making in non-culprit lesions whose severity is moderate (22), which contrasts with the recommendation to use FFR in intermediate stenoses in CCS (Class I) for patients with multi-vessel disease (MVD) (class IIa) (23).

## Does FFR Have a Role in STEMI?

FFR has no role in selecting the “culprit” vessel of ST elevation MI (STEMI), but it may be useful in assessing “bystander” stenoses. In COMPLETE, a landmark study of 4,041 STEMI patients with MVD, in which visual, rather than FFR guidance, was used, complete revascularization reduced the risk of cardiovascular deaths, MI and repeat revascularizations from 16.9 to 8.7% at 36 months when compared with a culprit-only-PCI approach; the benefit largely driven by a reduction in unplanned revascularization (24). In COMPARE-ACUTE (885 patients), DANAMI-3-PRIMULTI (627 patients) and FLOWER-MI (1,171 patients), an FFR-guided approach, rather than a purely visual one was used. In COMPARE-ACUTE, the primary outcome (composite of all-cause mortality, non-fatal MI, revascularisations and cerebrovascular events) occurred in 20% of the culprit-only revascularization group vs. 8% in the FFR-guided complete revascularization group (*p* < 0.001) (25). In DANAMI-3-PRIMULTI, the equivalent figures were 22 and 13%, respectively (*p* = 0.004) (26). The risk of future cardiovascular events was mainly driven by a 69% reduction in repeat revascularizations. In contrast, in FLOWER-MI, an FFR-guided approach in the non-culprit lesions in STEMI was not found to be superior to an angiography-guided strategy at reducing the risk of death, MI or repeat revascularization at 1 year. PCI of non-culprit lesions was performed in 66% of patients with the FFR-guided strategy and in 97% with the angiography-guided strategy. The primary outcome occurred in 5.5% (32 of 586 patients) in the FFR-guided approach vs. 4.2% (24 of 577 patients) in the angiographic-guided group (*p* = 0.31) (27). The difference was driven by a non-significant 77% higher risk of MI in patients assigned to the FFR group (18 patients in the FFR guided group vs. 10 patients in the angiographic guided group). The study was powered to detect a 37% lower risk of the primary composite outcome, but ultimately generated a wide confidence interval (hazard ratio, 1.32; 95% CI 0.78–2.23). In addition, intervention on the non-culprit lesions was encouraged to be performed at index presentation, rather than as a staged procedure. A larger RCT specifically addressing timing may be required. A parallel line of enquiry may be necessary to interrogate the hypothesis that conventional physiological assessment of bystander lesions may be of lesser importance than identifying vulnerable plaques.

## Why Do We Use so Little Physiology in the Acute Cardiac Catheter Laboratory?

Despite robust evidence supporting the use of FFR, in practise its use remains low, at <10% of PCIs, and in an even smaller proportion of diagnostic angiograms; the majority being in patients with CCS (2, 28). This low uptake in the acute setting may reflect the time and cost associated with deploying a pressure wire. Also, if stenting a borderline lesion is likely to be straightforward, it may be felt that a “quick fix” is reasonable. This is not, however, a position supported by the evidence. Other reasons for under-use may include complex anatomy, such as tortuosity, angulation, calcification and diffuse disease, in which manipulating a pressure wire might be hazardous. There may also be a lack of awareness of the accumulating evidence in ACS confounded by pressure on the operator to make a swift therapeutic decision in response to situational factors.

## Is FFR Reliable in Acute MI?

The validity of FFR in an acute MI has been questioned due to the possibility that blunted acute microvascular dysfunction might limit maximal hyperaemia, reducing the apparent physiological significance of a lesion (29). Does this mean that the FFR is “incorrect”? The value is indeed correct and reflects the current physiology, however the concern is that lesion significance may increase as the microvasculature recovers. Whilst this may be the case in a culprit lesion, in a study of 101 patients undergoing PCI for an acute MI (75 STEMI and 26 NSTEMI), the FFR measurements in 112 non-culprit vessels did not change between the acute presentation and follow up (30). In a study of 57 patients who had recovered from an MI an average 6 days previously, FFR < 0.75 was associated with sensitivity of 82% and specificity of 87% vs. SPECT imaging, and the relationship between the microvascular resistance in the infarcted territory and the viable myocardium was inversely proportioned (31). Similar results were found in a separate study of 48 patients (32).

## Problems With FFR in ACS

FFR is based upon the assumption that the relationship between flow and pressure in healthy and diseased arteries is predictable from a linear relationship. This is not strictly true, because energy is lost through friction in the diseased artery (viscous losses), and there is acceleration of flow at the outlet, producing a curvilinear relationship, which may particularly affect the acute patient (33). Also, the microvascular resistance, a crucial influence upon FFR, may differ between individuals, and be influenced by clinical, procedural, and extrinsic factors, which may be poorly controlled in the acute patient (34–36). Prior MI, diabetes, left ventricular hypertrophy, poorly controlled hypertension, endothelial dysfunction, and raised central venous pressure or left ventricular end-diastolic pressure (37) can also contribute. Uncertainty of the measurement itself is also a feature. The reproducibility of a therapeutic decision is >95% when the FFR is outside the 0.75–0.85 range, but only about 50% when it is close to 0.80 (38). This is of importance in the angiographic “borderline” lesion, for which the FFR is often also borderline. Other limitations are the lack of randomised data studying the use of FFR in left main stem disease, and technical difficulties measuring ostial, serial and bifurcation lesions (39). Measured FFR, therefore, has not achieved routine use in the management of ACS.

## What About CT and CT-FFR in ACS?

Functioning as a “triple rule-out,” CT can be used in the emergency department for patients with chest pain and no ECG changes to rule out severe coronary disease, acute aortic syndrome or pulmonary embolism (40). CT coronary angiography (CTCA) is useful at excluding significant CAD, with a negative predictive value (NPV) approaching 100% (41). In a study of 568 patients with suspected ACS, 84% were identified as low risk, discharged from hospital and had no adverse cardiac events at 30 days (42). In a study of 368 patients with chest pain and an inconclusive initial evaluation, CTCA showed that 50% of patients were free of CAD (43). Current ESC guidelines recommend CTCA as an alternative to invasive CAG in patients with low to intermediate risk of CAD and, when the troponin and ECG are inconclusive, to exclude ACS as class IIa (44). However, the RAPID-CTCA trial of 1,748 patients did not demonstrate a benefit of CTCA in reducing death, MI or stent thrombosis in suspected ACS when compared to standard practise (5.8 vs. 6.1%; *p* = 0.65) (45). CTCA is limited at predicting the haemodynamic significance of a lesion. CT-FFR, however, can demonstrate ischaemia-provoking lesions by modelling the coronary vasculature and incorporating computational fluid dynamics (CFD) (46) with a diagnostic accuracy of 81% (47, 48). CT-FFR is now recommended as an adjunct to CTCA in stable patients (49). It is limited, however, by the presence of calcification, tachycardia and arrhythmia (50). Other limitations are the availability of CT and, for CT-FFR, cost, and the requirement for off-site processing (up to 24 h). In the move towards timely interventional management for the ACS patient, CTCA is, therefore, generally impractical.

## What Is Virtual (v)FFR and How Is It Produced?

**Figure 2:**
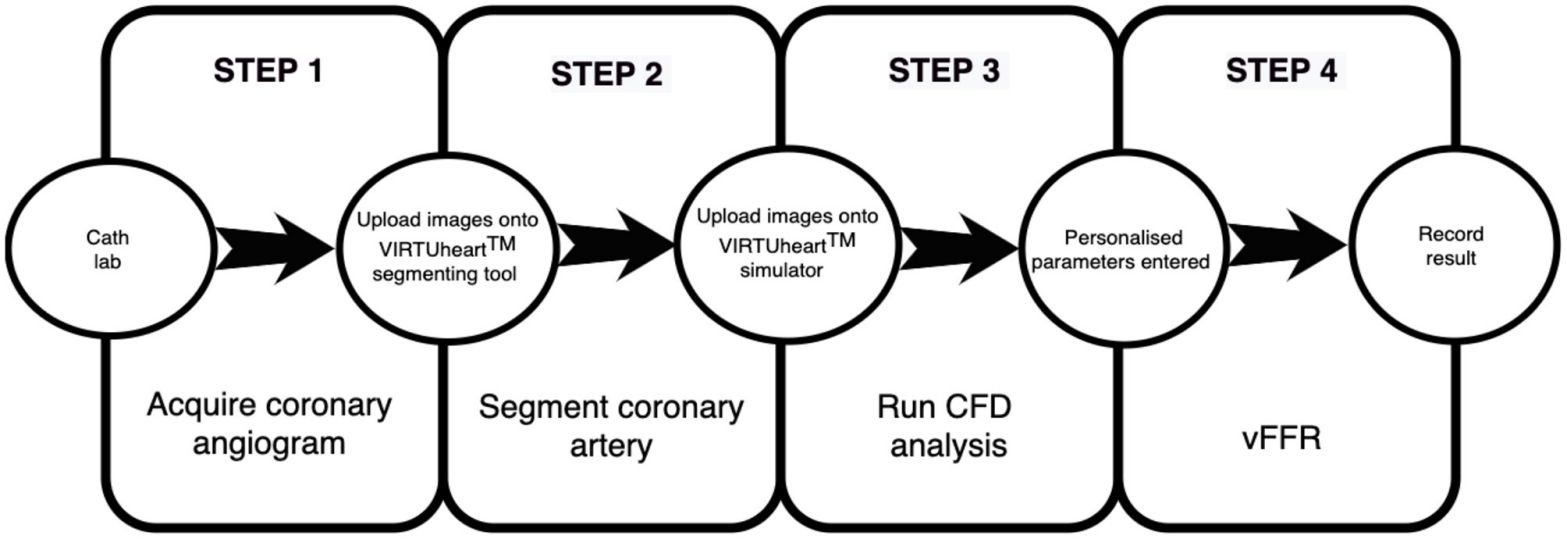
Summary of steps for vFFR calculation using the VIRTUheart™ software.

**Figure 3:**
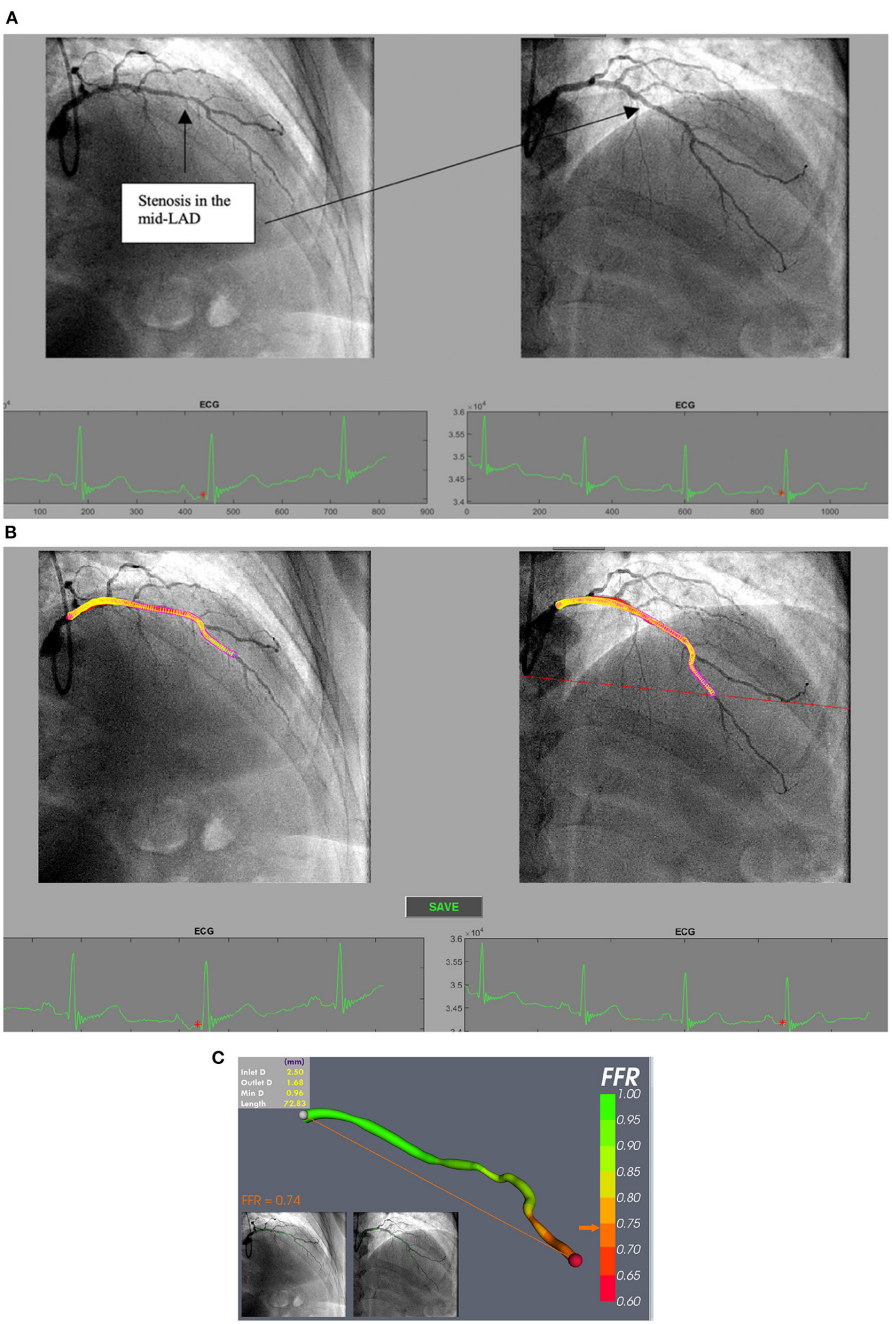
**(A)** Processing selected angiogram images. Two views (at least 30 degrees apart) of the LAD are chosen at end diastole (red dot on ECG tracing) from a patient with NSTE-ACS. **(B)** The LAD artery is now segmented and ready for a 3D reconstruction prior to CFD simulation. **(C)** vFFR result after 3D reconstruction and CFD simulation showing a vFFR in the LAD of 0.74.

vFFR is a novel technique to compute FFR non-invasively, without a pressure wire, using CAG images and CFD. CFD uses the physical laws of fluid flow, obeying the conservation of mass, momentum and energy, to simulate blood flow through a conduit. Several systems have been developed. The first, VIRTUheart™, has a high diagnostic accuracy (assessed against measured FFR) for detecting ischaemic lesions (FFR ≤ 0.80), with a computing time of <4 min (51, 52). The VIRTUheart™ software was initially validated against mFFR in the VIRTU1 study, which analysed 35 vessels in 19 patients, revealing an accuracy of 97% (51). In a second study larger study of 101 patients, VIRTUheart™ was found to have an accuracy of 92% (53). These are similar levels of accuracy compared with other vFFR systems, although there are no published head-to-head comparisons. The two key steps in using VIRTUheart™ are obtaining an accurate 3D model of the diseased coronary artery and estimating the microvascular resistance. The former requires a good quality angiogram, with optimal opacification, minimal magnification, no “panning” and minimal vessel overlap in at least two orthogonal planes at least 30 degrees apart, ideally with an ECG trace to identify an end-diastolic frame (54). Accuracy depends upon not only precise portrayal of the lesion, but also correct estimation of the MVR. In the absence of a wire measurement, MVR is estimated by either using a population average, or some form of personalisation. A typical process is shown in Figures 2, 3. The VIRTUheart™ system can calculate the vFFR between any points and diameters, show the percentage of stenosis and even offer a “virtual stents” (53).

## How Does the Different Software for Calculating Virtual FFR Differ From Each Other?

Several systems to calculate vFFR are available. Each has differing methodology. These include Quantitative Flow Ratio (QFR, Medis, Leiden, Netherlands and Pulse Medical Imaging, China) and Cardiovascular Angiographic Analysis System for Vessel FFR (CAAS-vFFR, Pie medical, Maastricht, Netherlands) based upon 3D quantitative coronary angiography (QCA); FFR_angio_ (Cathworks Ltd., Kfar-Saba, Israel) based upon 3D functional CA mapping with coronary rapid flow analysis; and Virtual Functional Assessment Index (vFAI) and Simplified Model of FFR Calculation (FFR_sim_) based upon 3D QCA and CFD. VIRTUheart™ is the Sheffield University system, currently confined to research use. QFR, FFR_angio_ and CAAS-vFFR are commercially available, with QFR being the first to obtain CE-mark and FDA approval. Whilst the first QFR study was based upon CFD, subsequent studies used faster computation using an algorithm incorporating coefficients from flow data to calculate pressure drops (55). QFR employs a 3D reconstruction and a QCA algorithm without reconstructing side branches (56). The software assumes that the coronary pressure remains constant in a normal coronary artery and that the distal coronary flow velocity is similar to the proximal. Based upon the mean hyperaemic velocities, the software can provide three different computation values: fixed-QFR (fQFR) based upon a flow velocity of 0.35 m/s; contrast-QFR (cQFR) applies Thrombolysis in Myocardial Infarction (TIMI) frame counting analysis at non-hyperaemic conditions; and adenosine-QFR (aQFR) uses intravenous administration of adenosine. FFR_angio_ provides colour-coded vFFR by applying a rapid analysis of flow based upon Poiseuille's law. A 3D coronary tree is generated and applies epipolar ray tracing with mathematical calculation. The software identifies the stenosis automatically by systematic segment, branch and junctional analysis. A user correction is required to correct any axis displacements contributed by movement. The resistance of the coronary arterial network in each segment is estimated by the vessel diameter and length, each vessel flow being based upon the overall impact of the resistance, and the FFR_angio_ value being calculated as the contribution of each narrowing to the total resistance and flow (57). CAAS-vFFR uses 3D model reconstruction, the vFFR being computed by measuring the pressure drop across a lesion by using simpler physical laws of viscous resistance and separation loss effects in coronary flow behaviour (58). In addition, it incorporates patient's specific aortic pressure with the assumption that the velocity of proximal coronary artery is preserved, along with the maximum hyperaemic blood flow previously determined from clinical data (59). Table 1 summarises the various CA-based FFR techniques.

**Table 1:** Summarising CA-based FFR software.

**Coronary angiography based FFR technique**					
**CA-based FFR technique**	**Company**	**Mathematical solution**	**Angiographic projections required**	**CA angle requirement**	**Key scientific reference**
vFFR (VIRTUheart™)	University of Sheffield	3D pseudotransient CFD based on Navier-Stokes equation	≥2 orthogonal images for each vessel	≥30 degrees	(51, 53)
QFR	Medis, Leiden, Netherlands and Pulse Medical Imaging, China	Analytical equations based on laws of Bernoulli and Poiseuille. Empiric flow velocity (fQFR), TIMI frame counting-derived contrast velocity at baseline (cQFR) and under hyperaemia (aQFR)	≥2 orthogonal images for each vessel	≥25 degrees	(55, 60, 60–62)
FFR_angio_	Cathworks Ltd., Kfar-Saba, Israel	Simple analytical equations based on Bernoulli and Poiseuille	≥2 orthogonal images for each vessel	≥30 degrees	(57, 63, 64)
CAAS-vFFR	Pie medical, Maastricht, The Netherlands	Simple analytical equations based on Bernoulli and Poiseuille	≥2 orthogonal images for each vessel	≥30 degrees	(59)
caFFR (FLASH FFR)	Rainmed Ltd., Suzhou, China	CFD based on post angiography TIMI frame counting of flow velocity	≥2 orthogonal images for each vessel	≥30 degrees	(65)
vFAI	Pie medical, Maastricht, The Netherlands	3D-QCA and steady state CFD	≥2 orthogonal images for each vessel	≥30 degrees	(66)

*CA, Coronary angiography; 3D, Three dimensional; QCA, Quantitative coronary angiography; VIRTUheart™, (University of Sheffield); QFR, Quantitative flow ratio (Medis, Leiden, Netherlands and Pulse Medical Imaging, China); FFRangio, 3D functional coronary angiography mapping with coronary flow analysis (Cathworks Ltd., Kfar-Saba, Israel); CAAS-vFFR, Cardiovascular Angiographic Analysis System (Pie medical, Maastricht, The Netherlands); caFFR, Coronary-angiography based FFR (FLASH software); vFAI, Virtual Functional Assessment Index*.

## What Is the Evidence for Virtual Coronary Physiology in ACS?

The main clinical hurdle for these virtual systems is to demonstrate accuracy against measured FFR (mFFR). This is usually expressed as the new system's percentage concordance with the treatment threshold (i.e., FFR> or <0.80). The QFR system, in a small study of 73 cases, disclosed accuracy of 88.3% (67) and in a larger study of 308 patients, ~90% (60). vFAI, which measures the average of the computed pressure ratio between distal and proximal vessel over a steady state CFD analysis has an accuracy of 88% (66). The accuracy of FFR_angio_ was 93% in a small study (63) and 87% in a larger study of 301 patients with FFR range of 0.75–0.85, which reflects the type of stenosis usually interrogated in real world setting (57). A sub-analysis from that study also demonstrated that FFR_angio_ is more accurate than other established FFR indices like instantaneous wave-free ratio (IFR) and diastolic hyperaemia-free ratio (DFR) (68). FFR_sim_, in a study of 68 vessels, disclosed an accuracy of 96% (69) which is similar to caFFR (65). The accuracy of CAAS-vFFR is 96% for vFFR measurements in their pre-defined “grey zone” (0.77–0.87) (59, 70). This technology also showed good correlation and accuracy with IVUS confirmed significant LMS disease in 147 patients with CAD (stable and unstable) (71). The accuracy of these systems vs. FFR in patients with ACS is displayed in Table 2.

**Table 2:** Summarising the evidence of vFFR in ACS.

**Summary of angiography based virtual FFR trials involving patients with ACS**															
**References**	**Software**	**Methods**	**Average processing time (min)**	**Total no of patients**	**ACS**	**NSTEMI**	**UA**	**Accuracy (%)**	**Correlation with FFR**	**BA agreement with FFR**	**Sen (%)**	**Spec (%)**	**PPV (%)**	**NPV (%)**	**AUC (%)**
Li et al. (65)	caFFR	Prospective, multi-centre, single-arm study	4.5 ± 1.5	328	275	-	275	95.7	0.89	±0.10	90.4	98.6	97.2	95	0.98
Tröbs et al. (72)	FFR_angio_	Retrospective analysis	n/a	73	22	4	18	90	0.85	±0.13	79	94	85	92	0.93
Fearon et al. (57)	FFR_angio_	Prospective, multi-centre, observational study	2.7	382	126	28	98	93	0.80	±0.14	93.5	91.2	89	94	0.94
Omori et al. (64)	FFR_angio_	Prospective, single-centre, single-arm study	9.6 ± 3.4	50	22	7	15	92.3	0.83	±0.14	92.4	92.4	n/a	n/a	0.92
Pellicano et al. (63)	FFR_angio_	Prospective, multi-centre, observational study	n/a	199	55	21	34	93	0.88	±0.10	88	95	n/a	n/a	0.80
Masdjedi et al. (59)	CAAS-vFFR	Retrospective, single-centre, observational study	n/a	100	40	26	14	n/a	0.89	±0.07	97	74	85	89	0.93
Tu et al. (55)	QFR	Prospective observational study	<10	68	9	-	9	88	0.81	±0.11	78	93	82	91	0.93
Xu et al. (60)	QFR	Prospective, multi-centre, observational study	n/a	308	66	-	66	92.7	0.86	±0.10	94.6	91.7	85.5	97.1	0.96
Westra et al. (61)	QFR	Prospective, observational investigator-initiated study	5	272	6	*	*	86.8	0.83	±0.12	86.5	86.9	76.4	93	0.92
Stähli et al. (62)	QFR	Single centre, retrospective study	n/a	436	123	18	105	93.4	0.82	±0.08	75	97.8	89.3	94.2	0.86
Papafaklis et al. (66)	vFAI	Retrospective study	n/a	120	41	8	33	90.4	0.78	±0.18	86.2	87.8	79.9	93.8	91.9

* *Not specified; n/a, not reported; BA, Bland-Altman; Sen, sensitivity; Spec, specificity; PPV, positive predictive value; NPV, negative predictive value; AUC, area under the receiver operating curve; caFFR, Coronary-angiography based FFR (FLASH software); FFRangio, 3D functional coronary angiography mapping with coronary flow analysis (Cathworks Ltd., Kfar-Saba, Israel); CAAS-vFFR, Cardiovascular Angiographic Analysis System (Pie medical, Maastricht, The Netherlands); QFR, Quantitative flow ratio (Medis, Leiden, Netherlands and Pulse Medical Imaging, China); vFAI, Virtual Functional Assessment Index*.

## What Are the Advantages of vFFR?

vFFR is fast. Computation time used to be the limiting factor, but now takes only minutes. The main time-limiting factor is manual image correction prior to the CFD step. The whole process can now be done in 'real time' in the acute CCL while the patient is on the table. It does not require a pressure wire or pharmacologic hyperaemia. In addition, the 3D anatomical model can assist with treatment planning, selection of stent size and 'virtual coronary intervention' together with an estimate of post-stent FFR (53, 63, 73). The same modelling technique can also predict the local haemodynamic consequence of a particular stenting strategy (74). Deploying vFFR does not supplant measured (m)FFR; if a lesion is equivocal at both angiography and vFFR, a pressure wire can still provide ultimate accuracy. There are, though, a few situations in which vFFR might actually be superior to mFFR. The first is serial lesions. Although a pressure wire “pullback” can provide some clues as to the relative significance of serial lesions, it is not infallible. In contrast, vFFR can reveal the FFR at each lesion simply by excluding the other lesion and modelling the lesion in question as if the other were not present. Of course, it can also model both together too. The second situation is when lesion complexity would make passing a pressure wire undesirable or hazardous. Another advantage is that vFFR can be used in any CCL without interventional capability. Also, the cost on a per-patient basis is likely to be low, because the business model for most commercially available systems is based upon an institutional licence.

## Does vFFR Have any Limitations?

Whilst the final coloured image appears seductive, its validity is mainly dependent upon good angiographic images, which require meticulous technique. Lesions at ostia, and at or close to the left main or a bifurcation, are difficult to model. In practise, in most CCLs, the radiographer is the most suitable professional to run the software but, even so, thorough training and practise is important. Casual users are considerably less accurate and consistent than regular users, largely due to errors in the 3D reconstruction; expert re-analysis of their models revealing errors that can lead to a change in the treatment decision in 37% cases (75). Although up to 50% of “standard” angiograms are unsuitable for processing, with a few simple improvements this proportion can be increased to 80% (76). This limitation is unfortunate, because it is the antithesis of measured FFR, where a scrupulous angiogram is less important. In addition, the distal outlet boundary condition is proximal to the CMV circulation, and a fundamental assumption of CMV function (maximal dilatation) is made to compute pressure from flow. The degree of CMV response to hyperaemia varies from person to person, which is why personalisation of this parameter is so important in vFFR. Also, very severe stenoses are difficult to model because the width of the lumen is less than a pixel, although in practise the likelihood is that such a lesion is physiologically significant. Finally, physiological measurement of all kinds is of most use in the assessment of angiographically intermediate lesions. So, however small the error on a vFFR system is, and it is usually at least ±0.10, if the vFFR is calculated to be 0.75–0.85, doubt will remain, and a measured value may be required. There is a further uncertainty, which also applies to measured FFR, which is that the physiological significance of a lesion, particularly in the acute patient, may not correlate with the presence of vulnerable plaque (77–79), probably explaining why long term outcomes are worse in ACS compared with CCS, even with physiological guidance. vFFR, therefore, whilst being an improvement over current management, is unlikely to provide a complete treatment strategy.

## Potential Application of vFFR in ACS

**Figure 4:**
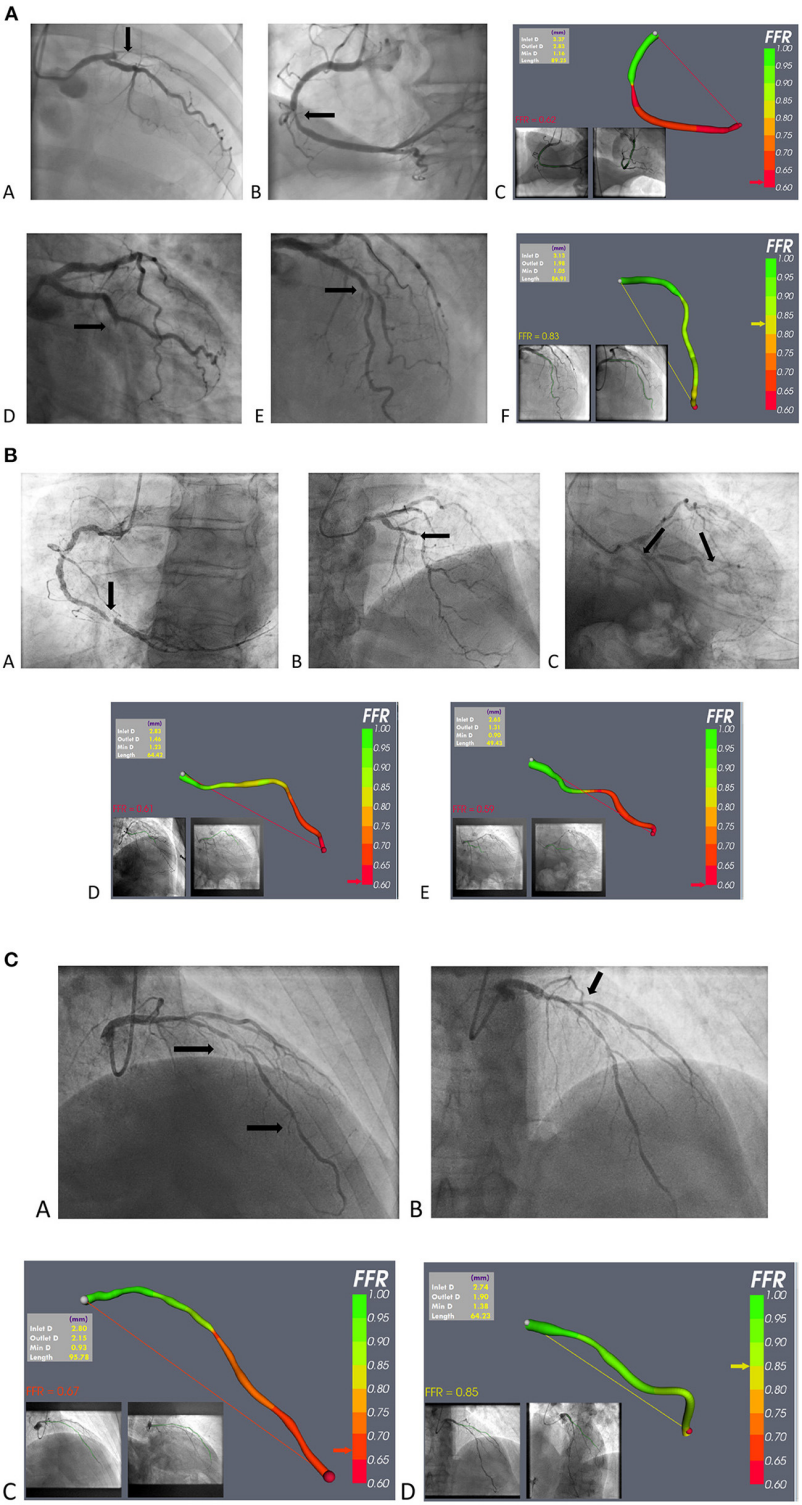
**(A)** Example of vFFR application in STEMI. (a–c) A case of anterior STEMI: (a) occluded proximal LAD; (b) mid RCA non-culprit stenosis; and (c) vFFR model of the RCA lesion. (d–f) A case of infero-lateral STEMI: (d) occluded mid Cx; (e) non-culprit mid-LAD stenosis; and (f) vFFR model of the mid LAD lesion. **(B)** vFFR use in NSTE-ACS; case 1. (a) Severe RCA stenosis, judged to be the “culprit,” and not requiring vFFR; (b) mid-LAD stenosis; (c) stenosis in the marginal branch (d) vFFR model of the LAD lesion; and (e) vFFR model of the marginal lesion. **(C)** vFFR use in NSTE-ACS; case 2. (a) Probable culprit LAD stenosis; (b) Probable bystander ostial diagonal stenosis; (c) vFFR model of the LAD lesion; (d) vFFR model of the D1 lesion.

A major attraction of vFFR for patients with ACS is that it can be used at the time of invasive management in a “one-stop shop,” in which coronary anatomy can be revealed alongside lesion-specific ischaemia testing. This is both time- and cost- efficient. It could be particularly useful in the common situation of multi-vessel, multi-lesion disease, when the culprit is frequently not angiographically obvious. Limited data support intervention for non-culprit lesions (14, 24–26) but, in the “real world” and, FFR guidance being rarely used (80), vFFR would provide an opportunity to select lesions requiring intervention without instrumentation and, perhaps more importantly, eliminating those that do not. This may be particularly important in apparent triple vessel disease, in which bypass surgery could be avoided. The greatest advantage is that vFFR could bring the advantage of coronary physiology to many more patients with ACS than at present. Finally, recent CFD-based modelling innovations are able to predict microvascular resistance which is known to be of prognostic significance in ACS (81). Examples of vFFR application in patients with ACS are shown in Figure 4.

## How Should vFFR Be Used in the Acute Cardiac Catheter Laboratory?

**Figure 5:**
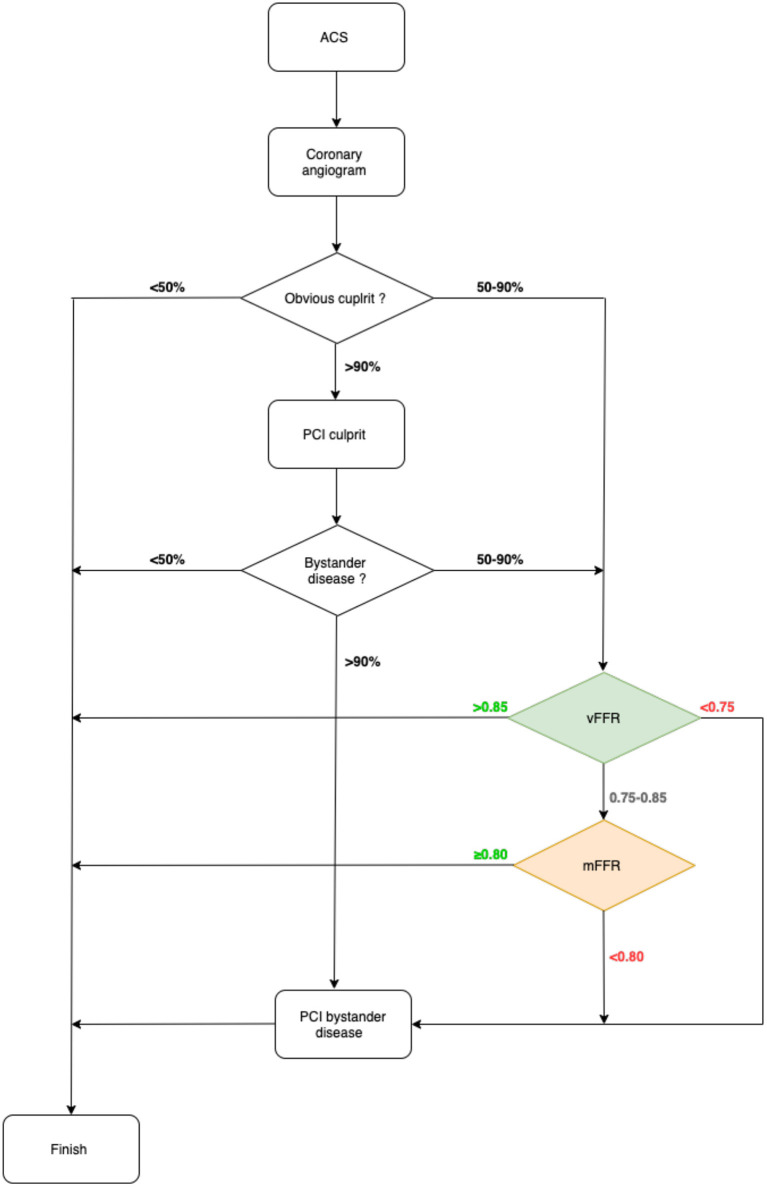
Proposed algorithm for the use of vFFR in the management of patients with ACS.

A potential algorithm for the invasive management of ACS, incorporating vFFR, is shown in Figure 5.

## The Integration of vFFR into Standard Management of ACS

Three approaches are possible. The first is simply to assume that the benefits seen in the trials of measured FFR are directly transferrable to vFFR and employ vFFR routinely. However, in the light of the limitations of vFFR outlined here, this assumption may be optimistic. The second would be to interrogate existing data derived from studies employing angiographic guidance, generating *post-hoc* vFFRs, and re-evaluating outcomes in accordance with vFFR. Because vFFR requires optimal angiographic images, however, many cases would be excluded using this approach; and it would be subject to the limitations of retrospective studies. The third would be to undertake prospective, randomised, controlled trials of vFFR- vs. CAG- guidance with clinical and health economic endpoints. A multi-centre RCT of 3,860 patients is currently investigating the superiority and efficacy of QFR- vs. CAG-guidance (82). Ultimately, endorsement in clinical guidelines will be required. Whichever approach is adopted, this technology is here to stay.

## Learning Points

1. Measured FFR is under-used for cost and logistic reasons.
2. Virtual (computed) FFR (vFFR) can be constructed from a coronary angiogram and does not need a pressure wire.
3. vFFR can be applied to intermediate lesions in ACS patients.
4. It can provide treatment guidance while the patient is in the cardiac catheter laboratory.
5. vFFR requires meticulous coronary angiography.

## Author Contributions

HH is supervised by JG, who leads the research team and designed the study. HH is responsible for conducting the research, recruiting patients, processing the cases, and analysing the data and results. VR, GP, and RH are responsible for coding, building the software as well as the main driving force behind the technological aspects, and improving the vFFR tool. MG, PDM, RG, GW, MM, TN, and PL contributed towards reviewing the paper with feedback and corrections prior to the final copy. All authors contributed to the article and approved the submitted version.

## Funding

HH, MG, GP, and RG were supported by the British Heart Foundation (TG/19/1/34451, FS/16/48/32306). PDM was supported by a Wellcome Trust Clinical Research Career Development Fellowship (214567/Z/18/Z). GW was supported by the Engineering and Physical Sciences Research Council (199760907). For the purpose of Open Access, the author has applied a CC BY public copyright license to any Author Accepted Manuscript version arising from this submission.

## Conflict of Interest

The authors declare that the research was conducted in the absence of any commercial or financial relationships that could be construed as a potential conflict of interest.

## Publisher's Note

All claims expressed in this article are solely those of the authors and do not necessarily represent those of their affiliated organizations, or those of the publisher, the editors and the reviewers. Any product that may be evaluated in this article, or claim that may be made by its manufacturer, is not guaranteed or endorsed by the publisher.

## Abbreviations

AUC: Area under the operator receiving curve
AHA: American heart association
ACS: Acute coronary syndrome
CABG: Coronary artery bypass grafting
CAD: Coronary artery disease
CAG: Coronary angiography
CAFFR: Coronary angiography based FFR
CAAS-vFFR: Cardiovascular angiographic analysis system for vessel FFR
CCL: Cardiac catheter laboratory
CCS: Chronic coronary syndrome
CFD: Computational fluid dynamics
CT: Computed tomography
CTCA: CT coronary angiography
CT-FFR: CT fractional flow reserve
COMPLETE: Complete revascularisation with multivessel PCI for myocardial infarction
COMPARE-ACUTE: Fractional flow reserve-guided multivessel angioplasty in myocardial infarction
DANAMI-3-PRIMULTI: Complete revascularisation vs. treatment of the culprit lesion only in patients with ST-segment elevation myocardial infarction and multivessel disease
ESC: European Society of Cardiology
FAME: Fractional flow reserve vs. angiography for multivessel evaluation
FFR: Fractional flow reserve
FFR_sim_: Simplified model of FFR calculation
FLOWER-MI: Multivessel PCI guided by FFR or angiography for MI
IFR: Instantaneous wave free ratio
MACE: Major adverse cardiovascular events
mFFR: Measured fractional flow reserve
MI: Myocardial infarction
MVD: Multi vessel disease
MVR: Microvascular resistance
NPV: Negative predictive value
NSTEMI: Non-ST elevation myocardial infarction
NSTE-ACS: Non-ST elevation acute coronary syndrome
PCI: Percutaneous coronary intervention
PPV: Positive predictive value
RCT: Randomised control trial
RFR: Resting full cycle ratio
STEMI: ST elevation myocardial infarction
TIMI: Thrombolysis in myocardial infarction
vFAI: Virtual functional assessment index
vFFR: Virtual fractional flow reserve
QALY: Quality adjusted life year
QCA: Quantitative coronary angiography
QFR: Quantitative flow ratio.
